# Trigeminal Herpes Zoster With Longitudinally Extensive Spinal Trigeminal Tract Involvement Presenting as an Isolated Facial Palsy Mimicking Ramsay Hunt Syndrome

**DOI:** 10.7759/cureus.111790

**Published:** 2026-06-30

**Authors:** Kumar Saurabh

**Affiliations:** 1 Department of Neurology, Orchid Medical Centre, Ranchi, IND

**Keywords:** lower motor neuron facial palsy, pcr-negative, ramsay hunt syndrome, spinal trigeminal tract, varicella-zoster virus

## Abstract

Varicella-zoster virus (VZV) is a neurotropic alpha herpesvirus capable of producing a wide spectrum of CNS disease, ranging from aseptic meningitis and vasculopathy to encephalitis and myelitis. Less widely appreciated is its capacity to extend centrally along the trigeminal pathway into the brainstem, producing intra-axial spinal trigeminal tract involvement that may remain clinically silent or manifest only as a cranial neuropathy, particularly when cerebrospinal fluid (CSF) polymerase chain reaction (PCR) is negative. We describe an immunocompetent 41-year-old man who presented with herpes zoster of the left hemiface in a maxillary-mandibular (V2-V3) distribution, followed by an acute left lower motor neuron facial palsy, initially indistinguishable from Ramsay Hunt syndrome. In the absence of encephalopathy, limb weakness, or cerebellar signs, magnetic resonance imaging of the brain demonstrated linear non-enhancing fluid-attenuated inversion recovery (FLAIR) hyperintensity extending across the posterior pons, medulla, and upper cervical cord, corresponding to the course of the spinal trigeminal tract and nucleus. CSF showed lymphocytic pleocytosis with otherwise unremarkable biochemistry; both herpes simplex virus and VZV PCR were negative. The patient was managed with intravenous acyclovir for 14 days, alongside short-course corticosteroids and supportive care. A transient rise in serum creatinine was managed with hydration and close monitoring while full-dose acyclovir was continued, without acyclovir-related neurotoxicity. Recovery was marked, with the House-Brackmann grade improving from V to III over follow-up and corresponding radiological regression of the brainstem signal change. The picture is best understood as sequential trigeminal-then-facial cranial neuropathy with longitudinally extensive spinal trigeminal tract involvement attributable to centripetal VZV spread, rather than a rhombencephalitic clinical syndrome. Such a combination -- trigeminal-territory zoster with longitudinally extensive spinal trigeminal tract involvement and an isolated facial palsy -- has only rarely been reported. This case illustrates the diagnostic dilemma of PCR-negative VZV CNS disease and underscores the value of clinicoradiological correlation, early antiviral therapy, and close neurological surveillance when a peripheral facial palsy emerges in the setting of cephalic herpes zoster.

## Introduction

Varicella-zoster virus (VZV) is a ubiquitous, neurotropic human alpha herpesvirus that establishes latency in cranial nerve, dorsal root, and autonomic ganglia after primary infection and may reactivate decades later to produce both peripheral and central neurological disease [1,2]. The neurological spectrum of VZV reactivation is broad and includes herpes zoster radiculopathy, post-herpetic neuralgia, cranial neuropathies, aseptic meningitis, meningoencephalitis, vasculopathy, and myelitis [2,3]. Among cranial nerve manifestations, the most readily recognized entity is Ramsay Hunt syndrome (herpes zoster oticus), classically characterized by the triad of ipsilateral peripheral facial nerve palsy, otalgia, and a vesicular eruption of the external auditory canal or hemiface, attributable to reactivation within the geniculate ganglion; brainstem involvement is uncommon.

VZV can extend from the trigeminal ganglion, centrally along the nerve root and the spinal trigeminal tract, into the brainstem, producing intra-axial signal change that ranges from clinically silent to symptomatic [1,2]. When such a brainstem inflammatory lesion is encountered, the differential is broad and carries direct therapeutic implications, encompassing infectious, autoimmune, and paraneoplastic disorders [4,5]. Among infectious etiologies, Listeria monocytogenes is the most frequently identified pathogen, followed by enteroviruses and the herpesviruses, of which herpes simplex virus (HSV) and VZV are reported less commonly [4-6]. Imaging characteristics carry significant discriminatory value: bacterial brainstem infection, particularly Listeria, typically produces ring-enhancing lesions or microabscesses, whereas viral inflammation more often demonstrates non-enhancing T2 or fluid-attenuated inversion recovery (FLAIR) hyperintensity without significant mass effect [6].

Diagnosis of VZV CNS infection conventionally rests on detection of viral DNA in CSF by polymerase chain reaction (PCR); however, PCR sensitivity is influenced by timing of sampling, prior antiviral exposure, and viral compartmentalization within neural tissue, and may therefore yield false-negative results in genuine cases [7,8]. In such situations, intrathecal anti-VZV IgG detection offers superior sensitivity but is not universally available in routine practice, so clinicoradiological correlation assumes a pivotal role in establishing a working diagnosis.

We present an immunocompetent adult who developed an isolated lower motor neuron (LMN) facial palsy on the background of cephalic herpes zoster, clinically indistinguishable from Ramsay Hunt syndrome, in whom neuroimaging unexpectedly revealed longitudinally extensive spinal trigeminal tract involvement within the brainstem despite negative HSV and VZV CSF PCR. To our knowledge, this convergence -- with parallel clinical and radiological response to acyclovir -- has only rarely been reported and underscores the diagnostic value of neuroimaging when CSF PCR is negative.

## Case presentation

A 41-year-old immunocompetent male presented with a 15-day history of a painful vesiculo-crusted eruption involving the left lower hemiface in a maxillary (V2) and mandibular (V3) trigeminal distribution; the ophthalmic (V1) division and periorbital region were spared. The eruption was accompanied from onset by ipsilateral otalgia, in keeping with involvement of the auriculotemporal (V3) branch of the trigeminal nerve; such trigeminal-territory ear pain can occur without auricular vesicles, as neuralgic pain and cutaneous eruption are dissociable. He had been initiated on subtherapeutic oral acyclovir (400 mg twice daily) along with topical therapy by a primary practitioner during the initial week of illness. Three days before admission, while the cutaneous lesions were crusting but not yet fully resolved, he developed deviation of the angle of the mouth to the right side, inability to fully close the left eye, and drooling of saliva, consistent with a left LMN facial palsy. The pre-existing otalgia did not appreciably change when the facial weakness appeared. There was no fever, headache, altered sensorium, seizure, vertigo, dysphagia, dysarthria, limb weakness, or gait disturbance at presentation.

On examination, he was conscious, oriented, and afebrile, with stable vital parameters. Neurological assessment confirmed an isolated left LMN facial palsy graded House-Brackmann V (asymmetry at rest with only barely perceptible voluntary motion -- loss of forehead wrinkling indicating frontalis weakness, incomplete eye closure, and slight movement at the angle of the mouth), with preserved corneal sensation and intact other cranial nerves, including hearing and vestibular function clinically (Figure 1). Motor power, deep tendon reflexes, plantar responses, sensory examination, cerebellar testing, and gait were all unremarkable. There were no meningeal signs.

**Figure 1:**
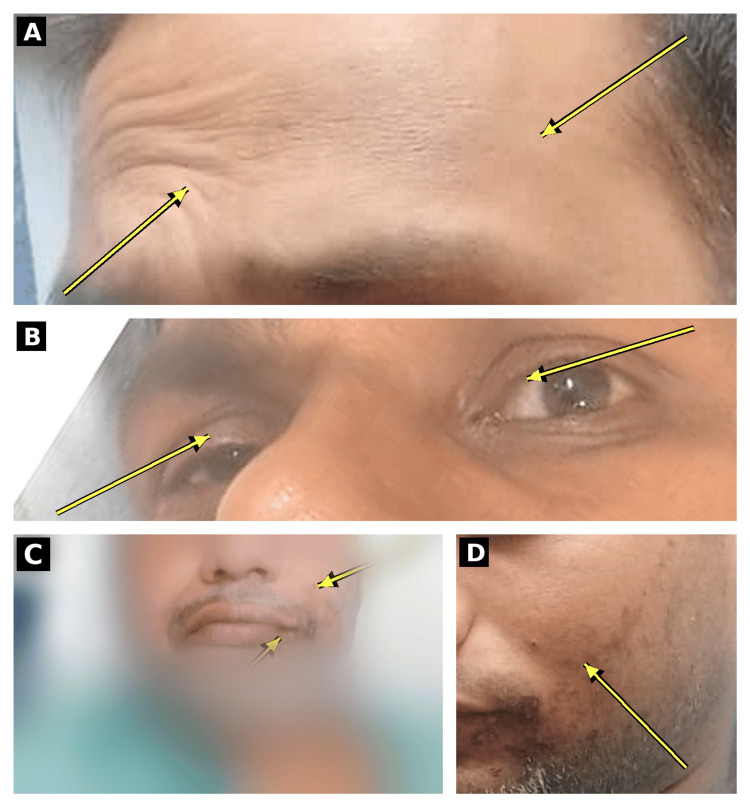
Clinical photographs demonstrating left lower motor neuron facial palsy with cutaneous herpes zoster. (A) Forehead: loss of wrinkling on the affected left side with preserved horizontal furrows on the normal right side, indicating frontalis weakness consistent with a lower motor neuron pattern. (B) Eyes: the affected left eye shows a wider palpebral aperture compared with the normal right eye. (C) Lower face: flattening of the left nasolabial fold with deviation of the angle of the mouth toward the unaffected right side. (D) Left lower face/cheek: crusted vesicular lesions in a maxillary-mandibular (V2-V3) distribution consistent with cutaneous herpes zoster

In view of the persistence of cutaneous lesions despite outpatient therapy and the new cranial nerve involvement, the patient was admitted for inpatient evaluation, escalation of antiviral therapy, and neuroimaging to exclude central involvement, given the recognized propensity of VZV to spread centrally from affected sensory ganglia to adjacent brainstem structures.

Magnetic resonance imaging (MRI) of the brain with contrast revealed a linear FLAIR hyperintensity involving the posterior pons extending into the medulla and upper cervical cord, without diffusion restriction, post-contrast enhancement, hemorrhage, mass effect, or abnormal cranial nerve enhancement. The radiological pattern was interpreted as consistent with intra-axial inflammation along the spinal trigeminal tract (Table 1, Figure 2). Cerebrospinal fluid (CSF) analysis demonstrated a slightly hazy fluid with a lymphocyte-predominant pleocytosis (20 cells/mm³, 95% lymphocytes), normal protein and glucose, and a low adenosine deaminase, supporting a viral pattern (Table 2). Multiplex PCR for HSV and VZV in CSF was negative. Blood and CSF bacterial cultures were sterile, arguing against Listeria. Routine blood investigations showed mild leukopenia and a mildly raised ESR (Table 3).

**Table 1:** Magnetic resonance imaging findings at presentation and on follow-up FLAIR, fluid-attenuated inversion recovery.

MRI	Findings
Initial MRI brain with contrast	Linear FLAIR hyperintensity involving the posterior pons extending into the medulla and upper cervical cord; no diffusion restriction; no contrast enhancement; no hemorrhage or mass effect; no abnormal cranial nerve enhancement. Impression: features suggestive of intra-axial inflammation along the spinal trigeminal tract
Follow-up MRI brain	Reduction in the previously noted brainstem FLAIR hyperintensity; no new lesions; no abnormal enhancement. Impression: radiological improvement consistent with clinical recovery

**Figure 2:**
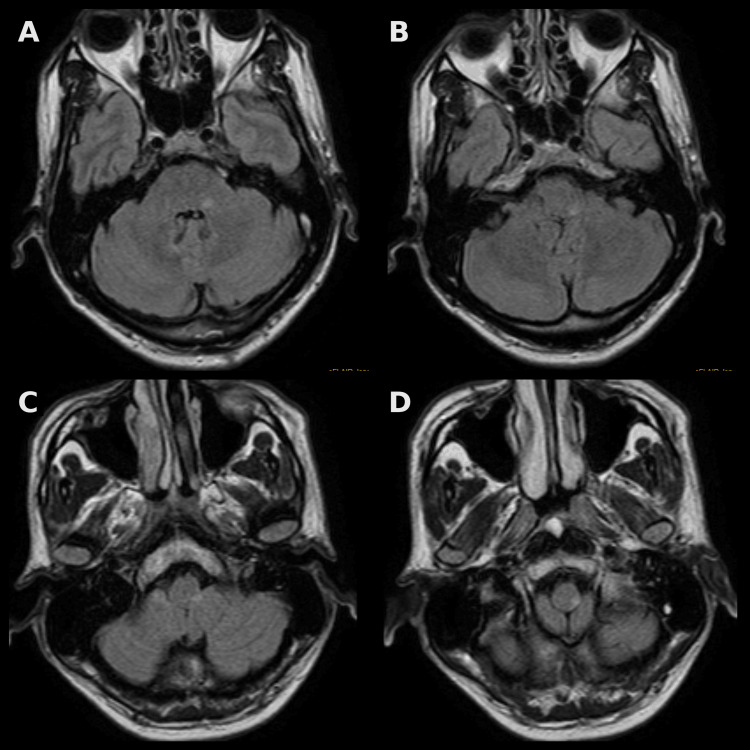
Serial axial FLAIR images demonstrating longitudinally extensive signal change along the spinal trigeminal tract. Axial FLAIR images arranged in a rostro-caudal sequence show a continuous, non-enhancing hyperintensity extending from the pons through the medulla into the upper cervical cord. (A) Mid-pons: ill-defined hyperintensity within the dorsal pontine tegmentum. (B) Lower pons/pontomedullary junction: caudal continuation of the signal abnormality. (C) Medulla: dorsal medullary hyperintensity. (D) Cervicomedullary junction: extension into the upper cervical cord. No diffusion restriction, contrast enhancement, hemorrhage, or mass effect was present. The pattern is consistent with viral (probable varicella-zoster virus) inflammation along the spinal trigeminal tract. FLAIR, fluid-attenuated inversion recovery.

**Table 2:** Cerebrospinal fluid analysis AFB, acid-fast bacilli; CSF, cerebrospinal fluid; HSV, herpes simplex virus; PCR, polymerase chain reaction; VZV, varicella-zoster virus.

Parameter	Result	Reference range	Interpretation
Appearance	Slightly hazy	Clear, colorless	Mild abnormality
Total leukocyte count	20 cells/mm³	0-5 cells/mm³	Pleocytosis
Differential count	Lymphocytes 95%, neutrophils 5%	Mononuclear predominant	Lymphocyte predominant (viral pattern)
Protein (microprotein)	17.3 mg/dL	15-45 mg/dL	Normal
Glucose (CSF)	102.1 mg/dL	40-70 mg/dL (≈⅔ of serum)	Not reduced
Adenosine deaminase	0.6 U/L	0-9 U/L	Normal; argues against tuberculosis
Gram stain	No organisms observed	No organisms	Negative
AFB stain	Not seen	Not seen	Negative for tuberculosis
India ink/negative staining	Negative for Cryptococcus	Negative	Negative
Cryptococcal antigen	Not detected	Not detected	Negative
Bacterial culture (aerobic)	No growth	No growth	Sterile
HSV 1/2 PCR	Not detected	Not detected	Negative
VZV PCR	Not detected	Not detected	Negative

**Table 3:** Blood investigations AKI, acute kidney injury; ALT, alanine aminotransferase; AST, aspartate aminotransferase; ESR, erythrocyte sedimentation rate; HBsAg, hepatitis B surface antigen; HbA1c, glycated hemoglobin; HCV, hepatitis C virus; HIV, human immunodeficiency virus; SGOT, serum glutamic-oxaloacetic transaminase; SGPT, serum glutamic-pyruvic transaminase.

Parameter	Result	Reference range	Interpretation
Hemoglobin	12.6 g/dL	13.0-17.0 g/dL	Mild anemia
Total leukocyte count	3.95 × 10³/µL	4.0-10.0 × 10³/µL	Leukopenia
Absolute lymphocyte count	0.89 × 10³/µL	1.0-3.0 × 10³/µL	Lymphopenia (viral pattern)
Platelet count	156 × 10³/µL	150-450 × 10³/µL	Normal
ESR	30 mm/h	0-10 mm/h	Mildly raised
Fasting plasma glucose	144.7 mg/dL	70-99 mg/dL	Transient hyperglycemia
HbA1c	4.8%	<5.7%	Non-diabetic
Serum sodium (nadir)	134.6 mmol/L	136-146 mmol/L	Transient mild hyponatremia
Serum potassium	4.13 mmol/L	3.5-5.1 mmol/L	Normal
Serum urea	27.9 mg/dL	17-43 mg/dL	Normal
Serum creatinine	0.93 (admission) ↑ 1.57 (peak) ↓ 1.02 (discharge)	0.72-1.18 mg/dL	Transient, reversible AKI (acyclovir/pre-renal); normalized
SGPT (ALT)	52 U/L	0-50 U/L	Mildly raised
SGOT (AST)	48 U/L	0-50 U/L	Normal
HIV/HBsAg/HCV	Non-reactive	Non-reactive	Immunocompetent
Blood culture (aerobic)	No growth	No growth	Sterile

A working diagnosis of probable VZV-associated spinal trigeminal tract involvement with secondary facial neuropathy was made despite the negative PCR. This rested on the clinicoradiological constellation: antecedent cephalic herpes zoster, an evolving LMN facial palsy, brainstem FLAIR hyperintensity in a pattern characteristic of viral inflammation, and CSF lymphocytic pleocytosis. The patient was commenced on intravenous acyclovir at 10 mg/kg every eight hours, alongside a short tapering course of corticosteroids, ocular protection with lubricants and taping for the left eye (for lagophthalmos), and supportive care. During treatment, serum creatinine rose transiently from 0.93 mg/dL at admission to a peak of 1.57 mg/dL over the first week of therapy. As the estimated creatinine clearance remained above 50 mL/min -- the threshold below which acyclovir dose reduction is recommended -- full therapeutic dosing (10 mg/kg every eight hours) was maintained with intravenous hydration, slow infusion, and close monitoring of renal function, rather than reducing the dose and risking subtherapeutic CNS exposure. Renal function recovered (creatinine: 1.02 mg/dL at discharge; Table 3), antiviral therapy was completed over a total of 14 days, and no acyclovir-related neurotoxicity -- such as encephalopathic features or myoclonus -- was observed.

The clinical course was favorable. Over the first week of therapy, the burning neuralgic pain regressed substantially, and facial weakness began to improve. By discharge, the House-Brackmann grade had improved by one level to grade IV, with reduced facial asymmetry at rest and early perceptible return of forehead movement, although eye closure remained incomplete and ocular protection was continued. A follow-up MRI of the brain demonstrated a reduction in the previously noted brainstem FLAIR hyperintensity, with no new lesions and abnormal enhancement, paralleling the clinical recovery (Table 1). At review approximately 10 days after discharge, the patient had regained complete eye closure with effort and showed further improvement to House-Brackmann grade III, with near symmetry at rest and forehead movement present. He remained hemodynamically stable throughout, without progression to encephalopathy, long-tract signs, or respiratory compromise, and was discharged on oral valacyclovir with structured neurological follow-up, ocular surveillance, and counseling to seek prompt reassessment for any new neurological symptom.

## Discussion

This case illustrates several conceptually important and pragmatically challenging facets of VZV-associated neurological disease: an apparent Ramsay Hunt presentation that belies underlying brainstem inflammation, the limitation of CSF PCR as a sole diagnostic gatekeeper, and the diagnostic and therapeutic utility of integrated clinicoradiological reasoning.

VZV exhibits a distinct predilection for neural tissue, with reactivation initiated within sensory ganglia and propagated centrally along nerve roots into adjacent brainstem structures [1,2]. In this patient, the cutaneous eruption lay in the maxillary-mandibular (V2-V3) trigeminal territory, and the longitudinally extensive lesion -- linear FLAIR hyperintensity extending from the posterior pons through the medulla into the upper cervical cord -- corresponds closely to the course of the spinal trigeminal tract and nucleus. Central propagation of VZV along the trigeminal pathway to produce such brainstem and cervicomedullary signal change is increasingly recognized: subclinical extension of viral inflammation into the brainstem and cervical cord was demonstrated on MRI in 10 of 16 patients with cranial or cervical zoster in one series [9], and a non-enhancing pontine trigeminal signal reflecting centripetal migration of virus from the trigeminal (Gasserian) ganglion has been described even in the setting of negative CSF viral studies [10]. The rostral extent of the lesion in the caudal pons lies immediately adjacent to the facial motor nucleus and fascicle, providing an anatomical explanation for the LMN-pattern facial palsy and for the clinical resemblance to Ramsay Hunt syndrome, despite the absence of the auricular (concha or external auditory canal) vesicles that characterize geniculate reactivation [11]. This sequential trigeminal-then-facial involvement, with lesions running along the spinal trigeminal tract and reaching the facial apparatus, is a recognized though uncommon pathway attributed to transaxonal or trans-synaptic spread of VZV across the brainstem between the fifth and seventh cranial nerves [10,12,13]. The picture is therefore best understood as trigeminal zoster with centripetal spread along the spinal trigeminal tract and secondary involvement of the adjacent facial nucleus or fascicle, producing a sequential facial palsy that mimics Ramsay Hunt syndrome rather than representing primary geniculate disease. The evidence for central involvement rests on the demonstrated intra-axial lesion rather than on the cutaneous picture: the absence of fresh auricular vesicles is consistent with a non-geniculate process but does not by itself localize the lesion, since VZV facial palsy may occur even without rash (zoster sine herpete). A contributory peripheral (perineural) component to the facial palsy cannot be excluded, but the anatomical correspondence between the lesion and the facial apparatus makes a central contribution at least equally plausible.

A brainstem inflammatory lesion carries a broad differential with direct therapeutic implications. Listeria monocytogenes, the most common infectious cause, typically produces a biphasic illness with cranial neuropathies, encephalopathy, and ring-enhancing or abscess-like lesions on MRI [4-6]. Autoimmune and paraneoplastic causes, including Behçet disease and antibody-mediated brainstem encephalitis, usually evolve over a longer course with distinct imaging signatures [5]. Against these, the absence of ring enhancement, the linear non-mass-forming FLAIR pattern, the lymphocyte-predominant CSF with normal protein and glucose, sterile blood and CSF cultures, and the antecedent dermatomal zoster favored a viral etiology. Among viruses, HSV characteristically involves the temporal lobes with seizures, cytomegalovirus produces a neutrophil-predominant pleocytosis largely in immunocompromised hosts, and enterovirus 71 predominantly affects children -- none of which fit this immunocompetent adult with lymphocytic CSF -- leaving VZV, on the background of cephalic zoster, as the most plausible candidate [4,6,14]. The CSF lymphocytic pleocytosis indicates active VZV-associated inflammation within the neuraxis but does not by itself localize the process or confirm the organism in the absence of a positive PCR or intrathecal anti-VZV antibody. In the absence of confirmatory virological or antibody testing, the diagnosis is best regarded as clinicoradiologically probable rather than definitive.

Although CSF PCR is regarded as the cornerstone of VZV CNS diagnosis, its sensitivity is not absolute: viral copy numbers vary and may be undetectable in active disease, particularly when sampling is delayed, when following antiviral therapy, or when inflammation is confined to the parenchyma rather than the ventricular CSF [3,7,8]. In a cohort of 20 patients with acute VZV encephalitis, PCR was negative in four, diagnosed instead on the basis of concurrent or recent zoster [8]. Intrathecal anti-VZV IgG, where available, complements PCR and may secure the diagnosis in PCR-negative cases; it was not accessible here, a real-world limitation that practicing neurologists must navigate.

The longitudinally extensive signal change, concurrent CSF lymphocytic pleocytosis, and treatment-associated radiological regression together indicate an active inflammatory process within the spinal trigeminal tract rather than an incidental subclinical imaging finding. Importantly, this constituted intra-axial tract involvement without a rhombencephalitic clinical syndrome: the patient had an isolated facial palsy with no encephalopathy, cerebellar, or long-tract signs. Longitudinally extensive involvement of the spinal trigeminal pathway is the less common pattern of zoster-associated brainstem disease, most reported lesions being focal and frequently subclinical [9]. Sequential trigeminal-then-facial involvement attributable to transaxonal or trans-synaptic VZV spread is a recognized phenomenon [10,12,13]: the nearest precedents are a maxillary-mandibular (V2-V3) zoster developing ipsilateral facial palsy with a focal medullary spinal trigeminal tract lesion [12] and a trigeminal zoster with a continuously long pons-to-cervical lesion accompanied by facial palsy [15], while a larger related literature describes spinal trigeminal tract and brainstem involvement arising from auricular (geniculate) Ramsay Hunt syndrome rather than trigeminal zoster [11,16,17]. Each of these differs from the present case in at least one respect -- auricular vesicles, hearing loss, positive VZV serology, an additional cranial-nerve palsy, or immunocompromise -- so that the specific combination reported here lacks an exact precedent. By contrast, the present case arose from trigeminal-territory (V2-V3) zoster, manifested as an isolated facial palsy, and was PCR-negative with clear radiological resolution. To our knowledge, the combination of trigeminal-territory zoster, an isolated PCR-negative facial palsy, and longitudinally extensive spinal trigeminal tract involvement with clean recovery has only rarely been reported; a picture dominated by an isolated facial palsy likewise remains uncommon within VZV CNS cohorts [3,18].

Current management of VZV CNS infection in immunocompetent adults centers on intravenous acyclovir at 10-15 mg/kg every 8 hours for 10-14 days, with extension reserved for severe or immunocompromised disease [3,19]. Because the present patient had objective intra-axial brainstem (spinal trigeminal tract) involvement accompanied by CSF lymphocytic pleocytosis -- that is, central rather than purely peripheral disease -- a full CNS-dose intravenous course was appropriate, in contrast to the oral antiviral regimen sufficient for uncomplicated zoster or geniculate Ramsay Hunt syndrome. Our patient achieved both clinical and radiological improvement on a standard 14-day intravenous regimen with short-course steroids, consistent with the favorable outcomes reported in immunocompetent VZV CNS disease when antivirals are initiated promptly [3,14,19]. Of note, during the cutaneous phase, the patient had received only subtherapeutic oral acyclovir (400 mg twice daily), well below the standard antiherpetic zoster regimen (for example, acyclovir 800 mg five times daily or valacyclovir 1 g three times daily); this likely permitted ongoing viral replication and may have facilitated centripetal spread to the brainstem. Blood and CSF bacterial cultures were negative, which -- together with the lymphocytic CSF profile, the non-enhancing imaging pattern, the immunocompetent host, and the absence of systemic features -- argued strongly against Listeria and other bacterial causes. Some limitations nonetheless remain: confirmatory intrathecal anti-VZV antibody testing and autoimmune or paraneoplastic antibody panels were not performed, so immune-mediated and other alternative etiologies could not be formally excluded, and follow-up was limited to the early post-discharge period of approximately 10 days, with longer-term outcome data unavailable. The case offers two clinically actionable lessons. First, in patients with cephalic herpes zoster who develop facial nerve palsy, a low threshold for neuroimaging is warranted because clinically unsuspected brainstem involvement may coexist with what otherwise resembles a purely peripheral process. Second, a negative CSF PCR should not be interpreted as definitive exclusion of VZV CNS infection; when imaging and CSF profile are consistent and an alternative etiology is not supported, empirical antiviral therapy and close neurological surveillance constitute the safer and more defensible course.

Taken together, this case adds to the limited literature on PCR-negative VZV CNS involvement and illustrates how an isolated facial palsy following cephalic herpes zoster may be the surface sign of central spinal trigeminal tract involvement, reinforcing the value of clinicoradiological diagnosis when laboratory confirmation is constrained.

## Conclusions

An apparent peripheral LMN facial palsy following cephalic herpes zoster may, on neuroimaging, prove to be the surface manifestation of an underlying central process. Central VZV involvement of this kind -- longitudinally extensive spinal trigeminal tract inflammation underlying a sequential facial palsy -- is uncommon but clinically important, and its recognition demands integration of clinical, biochemical, and radiological evidence rather than reliance on PCR alone. A negative CSF PCR does not exclude the diagnosis, particularly when antiviral therapy has been initiated, sampling is delayed, or the inflammatory focus lies within neural parenchyma. MRI is highly valuable both for establishing the diagnosis and for monitoring response. Early empirical intravenous acyclovir, supportive care, and close neurological surveillance for progression collectively offer the best prospect of recovery. The present case supports the view that clinicoradiological diagnosis can be a defensible strategy in selected cases of suspected PCR-negative VZV CNS infection.
